# Colon Targeted Guar Gum Compression Coated Tablets of Flurbiprofen: Formulation, Development, and Pharmacokinetics

**DOI:** 10.1155/2013/287919

**Published:** 2013-10-24

**Authors:** Sateesh Kumar Vemula, Vijaya Kumar Bontha

**Affiliations:** ^1^Department of Pharmaceutics, Chaitanya College of Pharmacy Education and Research, Kishanpura, Hanamkonda, Warangal, Andhra Pradesh 506001, India; ^2^Department of Pharmaceutics, Jangaon Institute of Pharmaceutical Sciences, Yeshwanthpur, Jangaon, Warangal, Andhra Pradesh 506167, India

## Abstract

The rationale of the present study is to formulate flurbiprofen colon targeted compression coated tablets using guar gum to improve the therapeutic efficacy by increasing drug levels in colon, and also to reduce the side effects in upper gastrointestinal tract. Direct compression method was used to prepare flurbiprofen core tablets, and they were compression coated with guar gum. Then the tablets were optimized with the support of *in vitro* dissolution studies, and further it was proved by pharmacokinetic studies. The optimized formulation (F4) showed almost complete drug release in the colon (99.86%) within 24 h without drug loss in the initial lag period of 5 h (only 6.84% drug release was observed during this period). The pharmacokinetic estimations proved the capability of guar gum compression coated tablets to achieve colon targeting. The *C*_max_ of colon targeted tablets was 11956.15 ng/mL at *T*_max_ of 10 h whereas it was 15677.52 ng/mL at 3 h in case of immediate release tablets. The area under the curve for the immediate release and compression coated tablets was 40385.78 and 78214.50 ng-h/mL and the mean resident time was 3.49 and 10.78 h, respectively. In conclusion, formulation of guar gum compression coated tablets was appropriate for colon targeting of flurbiprofen.

## 1. Introduction

Oral colon specific delivery of different drugs like anticancer drugs, anti-inflammatory drugs, antihelminthes, and proteins has gained increased importance from the last twenty years, to enhance the therapeutic benefits [1]. Development of colonic drug delivery is useful to treat local disorders of colon as well as to improve the delivery of proteins and peptides. Colon targeting is accomplished by using prodrug approach, pH-sensitive drug delivery, time-dependent delivery systems, and microbial degradation methods and formulated as tablets, capsules, multiparticulates, microspheres, and liposomes [2]. In these, microbial degradation method is the most popular and successful method to design the colon targeted formulations, which is capable of retarding the drug release in initial lag period (stomach and small intestine) and gives complete drug release in sustained manner within the colon [3].

Flurbiprofen (FLB) is a nonsteroidal anti-inflammatory drug used to treat inflammation and pain related to colon [4]. The frequent intake of FLB leads to gastric ulceration, bleeding, and other gastric complications [5]. Thus the development of colonic delivery of FLB is appropriate to reduce its side effects and achieve high local drug concentrations in the colon. Some of the recent research examples for FLB colon targeted systems are flurbiprofen microsponges [6], flurbiprofen pulsatile tablets [7], and flurbiprofen controlled release tablets [8].

Guar gum (GG) is a naturally occurring galactomannan polysaccharide obtained from the endosperm of the guar plant *Cyamopsis tetragonoloba,* which is a high molecular weight hydrocolloidal heteropolysaccharide composed of galactan and mannan units [9]. It has been suggested as a vehicle for oral controlled release purposes and for colon targeting due to its drug release retarding property and susceptibility to microbial degradation in the large intestine. Due to the slower rate of swelling, GG can be used to retard the release of drugs from tablets, and because of the polysaccharidic nature of GG, it is nondigestible by the human gut enzymes; however it can be degraded by the colonic microflora. Due to these advantages, GG is highly suitable for the delivery of drugs specifically to colon. GG is a widely used polymer in developing the colon specific drug delivery systems. Some of the research examples of drugs in colon targeted systems that utilized GG are tamoxifen [10], 5-aminosalicylic acid [11, 12], metoprolol tartrate [13], mebendazole [14], and trimetazidine dihydrochloride [15]. From the support of the above literature and information, it was planned to develop the FLB-GG colon targeted system to provide an effective and safe therapy. 

## 2. Materials and Methods

### 2.1. Materials

Flurbiprofen was a gift sample from FDC Limited, Mumbai, India. Guar gum and HPMC K4 M were gift samples from Matrix Laboratories, Hyderabad, India. All other chemicals used were of analytical grade. 

### 2.2. Preparation of Core and Compression Coated Tablets

Direct compression method was employed to prepare the core and compression coated tablets. Accurately weighed FLB and excipients, other than glidant and lubricant, were passed through 60 mesh sieve and mixed in a polybag for 5–10 min. The obtained blend was lubricated with talc and magnesium stearate for 5 min and compressed at 5000 kg compression force into tablets with 7 mm round flat punches using 16-station rotary tableting machine (Cmach, Ahmedabad, India; model no M/C CMB16STN, M/C NO 51/50). The amount of FLB was 100 mg and the final weight of the tablet was adjusted to 175 mg (Table 1). Then the core tablets were compression coated with different compositions of coats given in Table 2 using the procedure given in Vemula and Veerareddy, 2012, with 10 mm round, flat, and plain punches [8].

### 2.3. Evaluation of Physical Parameters

The prepared compression coated tablets were evaluated for weight variation, hardness, friability, and drug content. To estimate the weight variation, 20 tablets from each formulation were weighed using an electronic weighing balance (Shimadzu, Japan) and calculated the average weight and deviation. The hardness of six tablets was determined using Monsanto tablet hardness tester. Friability was measured on ten tablets in a Roche friabilator (Electrolab, India). To estimate the drug content, ten tablets were crushed, and powder equivalent to 100 mg of FLB was weighed accurately, prepared the solution, and analyzed for FLB using the HPLC method using UV detector at wavelength of 254 nm.

### 2.4. *In Vitro* Dissolution Study

The *in vitro* dissolution study for compression coated tablets was carried out using USP XXIV Type I dissolution apparatus (Electro lab, TDT-08L) at 37 ± 0.5°C temperature and 50 rpm rotation speed. To simulate the gastrointestinal transit conditions, the tablets were subjected to different dissolution media. Initially, the drug release was carried out for 2 h in simulated gastric fluid (SGF, pH 1.2), then in enzyme-free simulated intestinal fluid (SIF, pH 7.4) for 3 h, the average small intestinal transit time, and finally in simulated colonic fluid (SCF), that is, pH 6.8, phosphate buffer containing 4% w/v of rat caecal contents up to 24 h to mimic colonic conditions. At specific time intervals, 5 mL of the samples were withdrawn, filtered, and analyzed for FLB. 

### 2.5. *In Vitro* Release Kinetics

The obtained data from *in vitro* dissolution studies were fitted to zero-order, first-order, and Higuchi models to elucidate the drug release pattern and mechanism from the compression coated tablets [16]. Korsmeyer-Peppas model is used to explain the mechanism of drug release from the above tablet formulations [17]. The mean dissolution time (MDT) is defined as the sum of different release fraction periods (release areas) during dissolution studies divided by the initial loading dose and is calculated [18]. T10% and T80% (time in hours to take 10% and 80% drug release, resp.) were calculated to clarify the colon specific release from FLB-GG compression coated tablets [19].

### 2.6. Stability Studies

To evaluate the stability of FLB in compression coated tablets, the stability studies were carried out according to ICH guidelines. In this study, optimized formulation F4 was sealed in aluminum packaging coated inside with polyethylene, and three replicates were kept in the humidity chamber maintained at 40 ± 2°C and 75 ± 5% RH for six months [20]. Samples were collected after six months of storage and analyzed for the drug content and *in vitro* dissolution rate [21]. Then the data was statistically analyzed using paired *t*-test to test the significance of difference at 0.05 level of significance. Finally, the similarity index was calculated between dissolution rates of optimized tablets before and after storage to prove the stability of dosage form.

### 2.7. *In Vivo* Study in Healthy Volunteers

A crossover design was followed in the present study, in which twelve human volunteers were used and separated into two groups. In the first phase of study group I volunteers (*n* = 6) received an immediate release core tablet (dose 100 mg) whereas group II (*n* = 6) volunteers received colon targeted compression coated tablet (dose 100 mg). Whereas, in the second phase of study, after ten-day washout period, group I volunteers received colon targeted compression coated tablet, group II volunteers received immediate release core tablet. All the volunteers received the tablets on an empty stomach with sufficient water, and then a standard breakfast was provided after 2 h of the study. At regular time intervals lunch and dinner were provided in standard quantity. In both cases, the blood samples were collected at 0, 0.5, 1, 2, 3, 4, 5, 6, 8, 10, 12, 18, and 24 h in vials. The institutional ethical committee (approval no. 338-06/JIPS/JNG/IHEC/2011) approved the present protocol. 

### 2.8. HPLC Analysis of Plasma Samples

To analyze the plasma samples, the method developed by Veerareddy and Vemula, 2012, was used in the present study. The collected blood samples were centrifuged at 4000 rpm for 15 min, and the plasma was separated and transferred to 5 mL microcentrifuge tubes. To the 1 mL of the above plasma, 1 mL of acetonitrile was added and centrifuged for 10 min at 3000 rpm, and the supernatant liquid was separated and stored at −40°C until the analysis of sample for unchanged drug. Then the quantitative determination of FLB in human plasma was performed using HPLC method by injecting the supernatant liquid after diluting it into the HPLC column (loop volume 20 *μ*L and flow rate 1 mL/min). The analysis was performed at ambient temperature, the run time was set to 8 min, and the eluents were monitored at 254 nm using UV detector. The standard curve was constructed using standard drug solutions ranging from 200 to 1000 ng/mL by the above method and was used to estimate FLB in human plasma [7]. 

### 2.9. Pharmacokinetic Analysis

All the possible and required pharmacokinetic parameters were calculated using FLB plasma concentration-time data. From the time versus plasma concentration graph, the peak plasma concentration (*C*_max_) and the time to reach peak plasma levels (*T*_max_) were obtained. Other pharmacokinetic parameters were calculated using Kinetica software (Kinetica 2000 version 3.0, InnaPhase Corporation, 2000). From linear part in the elimination phase of a semilog plot of concentration versus time, the elimination rate constant (*ke*) was calculated. The area under the concentration versus time curve (AUC) from 0 to *t* h was calculated by applying the trapezoidal rule, and then the AUC extended to infinity (0 to ∞ h) that represents the extent of bioavailability of FLB. The area under first moment curve (AUMC) was obtained from the plot of product of plasma drug concentration and time versus time. The AUMC_0–*t*_ and AUMC_0–*∞*_ were calculated by trapezoidal rule. The mean residence time (MRT) is defined as the time needed for 63.2% of drug being eliminated from the body under constant clearance conditions, and it was calculated using AUC and AUMC.

### 2.10. Statistical Analysis

The estimated pharmacokinetic parameters of both immediate release and colon targeted tablets of FLB were subjected to statistical analysis using analysis of variance (ANOVA) to test the significance of difference. A value of *P* < 0.05 was considered statistically significant. 

### 2.11. *In Vitro*-*In Vivo* Correlation (IVIVC)

The IVIVC of dissolution rate and the absorption rate has been widely used in the quality control and formulation development of oral formulations. In the present study the *in vitro* cumulative percent of FLB release of optimized formulation was compared against the extent of absorption, that is, cumulative AUC values of the same formulation.

## 3. Results

### 3.1. Evaluation of Physical Parameters

All the physical properties of FLB-GG compression coated tablets were given in Table 3. From the weight variation test, it was found that the weight variation of the tablets was in the range of 377.55 ± 3.43 mg–371.04 ± 4.63 mg. The pharmacopoeial limit for weight variation in all the tablets should not be more than 5% of the average weight. The hardness of the tablets was found to be in the range of 6.43 ± 0.35 kg/cm^2^–6.21 ± 0.12 kg/cm^2^. The percentage friability for all formulations was below 1%, that is, 0.09%–0.18%, indicating that the friability is within the prescribed limits. The tablets were found to contain 100.87 ± 1.15%–98.83 ± 1.46% of the labeled amount indicating uniformity of drug content.

### 3.2. *In Vitro* Dissolution Study

**Figure 1:**
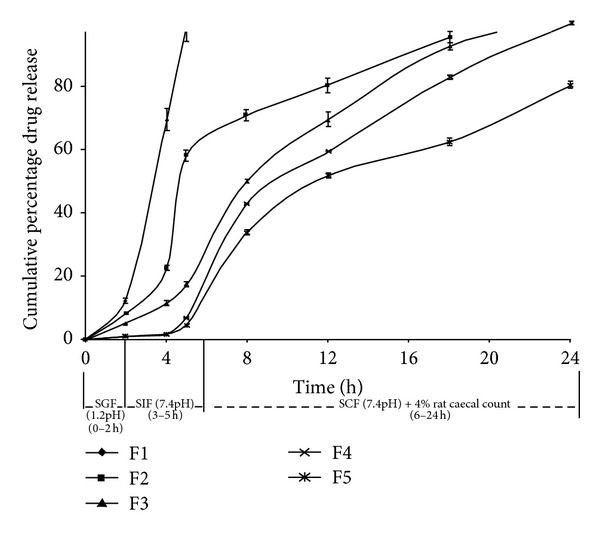
Release profile of FLB from compression coated tablets.

From the dissolution study of core tablets in 6.8 pH phosphate buffer, it was found that 99.59 ± 40.69% drug released in 15 min. In the preliminary studies, the compression coat weight was optimized by conducting the *in vitro* drug release studies for the formulations containing different coat weights (150 mg, 175 mg, 200 mg, 225 mg, and 250 mg). From this, 200 mg was found as suitable weight to give the protection to inner core tablets. So for the formulations F1–F5, the compression coat weight was set as 200 mg.  Figure 1 showed the release profiles of FLB from the GG compression coated tablets of varying amounts (F1–F5), and it was found to vary from 4.59 ± 0.06 to 99.25 ± 0.28% after 5 h of testing in simulated gastric and intestinal fluids, and the percent of drug release was increased gradually after 5 h, and it was found to be 80.25 ± 0.68 to 101.23 ± 1.96% in 24 h testing in simulated colonic fluid. From the results of *in vitro* drug release studies, the cumulative mean percent of FLB released from compression coated tablets containing varying amounts of GG (20 mg, 40 mg, 60 mg, 80 mg and 100 mg), with incorporation of 80 mg of polymer in the total tablet weight (F4), was found to be satisfactory to formulate a tablet with good integrity and satisfactory *in vitro* drug release.

### 3.3. *In Vitro* Release Kinetics

From the values of *K* and *r* ^2^ (correlation coefficient of the regression analysis) of zero-order, first-order, and Higuchi models of designed formulations, it was found that the compression coated tablets showed the highest values for zero-order model (Table 4). From the Peppas model, the *n* values calculated for different formulations were found in the range of 1.1592 to 3.3104. The MDT values were found to be 2.35–13.71. The T10% and T80% values of optimized formulation (F4) were found to be 5.6 h and 17.9 h, respectively. All these results were given in Table 5.

### 3.4. Stability Studies

In consideration of the potential utility of the formulation, stability studies were carried out at 40 ± 2°C and 75 ± 5% RH for six months to assess their stability. After storage for six months, the tablets were subjected to drug assay and *in vitro* dissolution studies (Table 6), and from the statistical analysis there was no significant difference between before and after storage (*P* < 0.05). The similarity index value between dissolution profiles of optimized formulation before and after storage was found to be 86.82.

### 3.5. Pharmacokinetics in Healthy Volunteers

**Figure 2:**
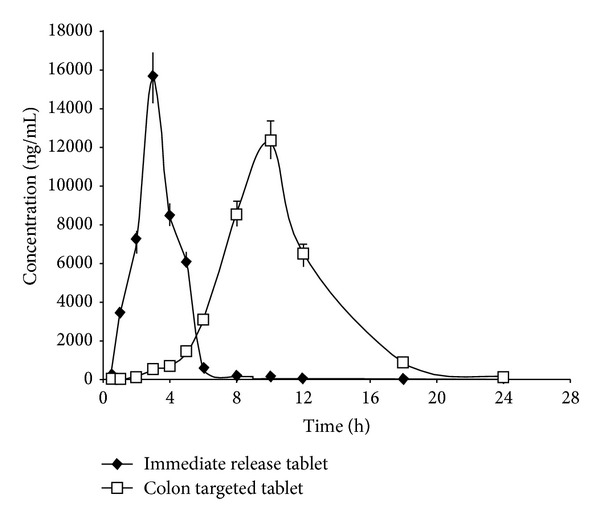
Time versus mean plasma concentration profiles of FLB following the oral administration of colon targeted compression coated tablet F4 and immediate release tablet in human volunteers (*n* = 6).

In this present study, pharmacokinetic evaluation was done on colon targeted compression coated tablets F4 in comparison to immediate release tablet of FLB. The mean FLB plasma concentrations of six human volunteers following the oral administration of colon targeted compression coated and immediate release tablets were shown in Figure 2, and the mean pharmacokinetic parameters from the *in vivo* experiments of both tablets were given in Table 7.

### 3.6. *In Vitro*-*In Vivo* Correlation (IVIVC)

**Figure 3:**
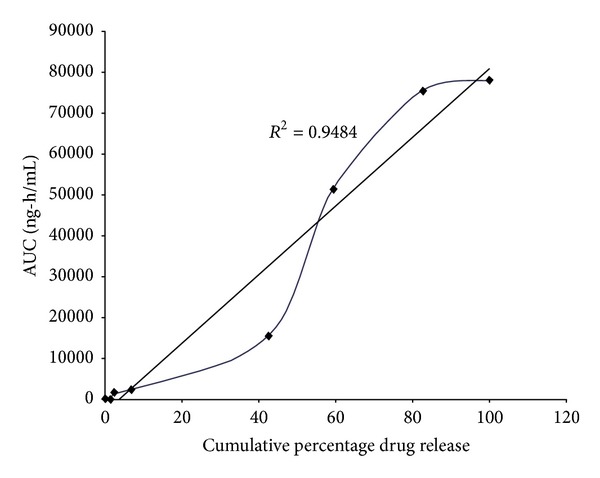
*In vitro*-*in vivo* correlation plot of colon targeted formulation F4.

IVIVC was carried out for optimized formulation F4 by plotting the *in vitro* cumulative percent of FLB release on *x*-axis and the cumulative AUC obtained after oral administration on *y*-axis (Figure 3). From the above plot it was observed that there was a good correlation with correlation coefficient *r* ^2^ = 0.9484 indicating better relationship between the *in vitro* cumulative percent of FLB release and the *in vivo* drug absorbed (AUC).

## 4. Discussion

The intention of present investigation is to develop the flurbiprofen colon specific delivery to facilitate the maximum drug delivery at the required site to gain the therapeutic benefit. In this study, guar gum compression coated tablets were prepared to achieve the colon specific release of FLB and evaluated to prove the colon specificity. Weight variation, thickness, hardness, friability, and drug content of all the tablet formulations were complied with pharmacopoeial standards, so all the tablets were with acceptable physical characteristics. In weight variation test, the pharmacopoeial limit for tablets is not more than 5% of the average weight. The average percentage deviation of all tablet formulations was found to be within the specified limit, and hence all the formulations passed the uniformity of weight as per the official requirements of Indian Pharmacopoeia, 1996. From the physical characterization, all the tablet formulations were uniform in hardness, friability, and drug content uniformity. 

The optimized compression coat weight for the better drug release profiles suitable for colon specific release of FLB was studied by formulating the compression coated tablets with different coat weights. From the dissolution study, it was found that 200 mg is the suitable compression coat weight for colon targeting. The cumulative mean percent of FLB released from compression coated tablets containing varying amounts of GG was estimated to optimize the amount of polymer suitable to produce colon specific drug delivery. The formulation containing 80 mg of GG was believed to be better among other formulations to produce colon targeted drug delivery of FLB. In a study reported in the literature, 125 mg of GG in the coat weight of 175 mg showed similar types of results for indomethacin [22]. In our study, 80 mg of GG in the coat weight of 200 mg showed similar results.

*In vitro *dissolution study of F1–F5 formulations show the effect of GG amount on drug release from the compression coated tablets (Figure 1). A minimal amount of the drug (<10%) is released in the physiological environment of stomach and small intestine, and progressive drug release was observed in the colonic region with 80 mg of GG. From these results, the formulation F4 is considered as the optimized formulation, which showed 6.84% drug release in the initial lag period (5 h) followed by 99.86% drug release for 24 h in a sustained fashion. Formulations with higher than 80 mg of GG showed negligible drug release in the initial lag time but failed to complete the drug release in 24 h. In the present investigation, 10% HPMC was added in all the formulations to improve the mechanical strength of tablets, because of low compressibility of GG alone. Similar types of observations were observed in study developed by Veerareddy and Manthri, that is, guar gum compression coated tablets of piroxicam [23]. In summary, the formulation F4 showed less than 10% drug release in 5 h, and it was progressively increased to 100% in 24 h which indicates that only a small amount of drug was released in stomach and small intestine, and a large amount of dose was released in colonic environment due to microbial degradation of GG. Thus the formulation F4 was selected as the optimized one for further pharmacokinetic evaluation.

The drug release kinetics showed high correlation coefficient values for zeroorder than first order indicating that the drug release from compression coated tablets followed zero-order patterns. Zero-order release was also observed in a study with flurbiprofen using HPMC in the compression coat [8]. The high regression value of Higuchi model ensured the release of drug from compression coated tablets followed by diffusion mechanism. The *n* values are calculated for different formulations indicating a supercase-II transport. The MDT was higher when increasing the polymer content indicating better controlled release *time* in hours to take 10% and 80% drug release (T10% and T80%) which explained the ability of colon specific release from compression coated tablets. From these parameters formulation F4 showed 5 h lag time and also gave the complete drug release in colon when compared to other formulations. After storage for six months, the formulation was subjected to a drug assay, and *in vitro* dissolution studies and the data showed that there was no significant change in formulation in the sense of drug content and dissolution behavior. The similarity index value was found to be 86.82, which is more than 50 indicateing similarity between the dissolution profile before and after storage.

The *in vitro* drug release studies of GG compression coated colon targeted tablets of FLB (F4) revealed that they give considerable amount of drug release in the colon without loss in the upper gastrointestinal tract. Further the pharmacokinetic evaluation in healthy volunteers is required to prove the capacity of colon specific release of FLB. From the evaluation, *T*_max_ represents rate of absorption and AUC is related to extent of absorption while *C*_max_ is related to both. MRT gives the tendency of drug to remain in the body. From the pharmacokinetic evaluation, after oral administration of immediate release tablet, FLB appeared almost immediately within 30 min while it required about 5 h in case of colon targeted compression coated tablets to make the drug appear significantly in plasma. The immediate release (core) tablets disintegrated very fast in GI tract and resulted in quick absorption of the drug from upper GIT and producing peak plasma concentration *C*_max_ of 15677.52 ng/mL at 3 h *T*_max_. On oral administration of colon targeted tablets (F4), FLB reached peak concentration (*C*_max_ = 11956.15 ng/mL) at 10 h *T*_max_ which revealed that the colon targeted tablet did not allow the release of FLB in upper GIT. The shift in the *T*_max_ to a higher value is typical for the colon targeted drug delivery systems [24]. But after reaching the colonic environment, drug is released progressively from the colon targeted tablet by the microbial enzymatic action on the GG tablets [25]. The area under the curve (AUC) for the immediate release and compression coated colon targeted tablets of FLB was 40385.78 and 78214.50 ng-h/mL, respectively. These results showed that the colon targeted compression coated tablets did not release the drug appreciably in upper GIT but released it slowly and progressively in the colon. The MRT of immediate release and compression coated colon targeted tablets of FLB was 3.49 h and 10.78 h, respectively, demonstrating long resident time for colon targeted tablet when compared to immediate release tablet.

The statistical analysis of pharmacokinetic parameters of immediate and colon release tablets was performed by ANOVA test. From the results there was a significant difference in the *C*_max_ between immediate release and colon targeted tablets, demonstrating that colon targeted tablets did not release the FLB in upper GIT. Similar types of results were observed in guar gum based colon targeted tablets of mebendazole [26]. The *T*_max_, AUC, and MRT of immediate release tablets were significantly different from colon targeted compression coated tablets representing delayed release of FLB specifically to colon in slow manner. From the IVIVC results, there was a good correlation between the *in vitro* and *in vivo* parameters. From all these observations it was concluded that the colon targeted GG compression coated tablets showed negligible FLB release in stomach and small intestine but are released significantly in colon.

## 5. Conclusion

In the present study, an attempt was made to develop the guar gum compression coated tablets of FLB to produce the colon specific release without loss in the upper GIT. From the *in vitro* drug release studies, F4 formulation showed significant level of drug release in the colon with negligible release in the first 5 h. The drug release from these tablets showed zero-order profile, and the drug release mechanism was supercase-II transport. The accelerated stability studies proved the stability of GG compression coated tablets. The estimated pharmacokinetic parameters showed that the colon targeted tablets did not release the drug in stomach and small intestine but released in the colon when compared to immediate release tablets. In conclusion, development of GG compression coated tablets based on microbial-dependent method is a good approach for colon targeting of FLB.

## Figures and Tables

**Table 1:** Composition and characterization of FLB core tablets

Ingredients	Quantity (mg)	Core tablet evaluation parameters	*n*	Observed values
Flurbiprofen	100	Weight variation (mg)	20	174.85 ± 2.92
Spray dried lactose	50.5	Core thickness (mm)	20	3.01 ± 0.02
Crospovidone	17.5	Core diameter (mm)	20	7.04 ± 0.01
Sodium lauryl sulphate	1.75	Hardness (kg/cm^2^)	6	2.83 ± 0.35
Talc	3.5	Friability (%)	10	0.58
Magnesium stearate	1.75	Disintegration time (sec)	3	35.67 ± 3.51
Core weight	175	Content uniformity (%)	3	100.28 ± 1.12
		% drug release in 15 min (*Q*_15_)	3	99.59 ± 0.69

**Table 2:** Composition of FLB compression coated tablets.

Formulation code*	FLB core tablet (mg)	Guar gum (mg)	Total tablet weight (mg)
F1	175	20	375
F2	175	40	375
F3	175	60	375
F4	175	80	375
F5	175	100	375
Total thickness	3.68 ± 0.04 mm		
Total diameter	10.04 ± 0.02 mm		
Coat thickness	0.62 ± 0.05 mm		
Coat diameter	3.04 ± 0.01 mm		

*Each compression coat formulation contains 10% HPMC, 1% magnesium stearate, 2% talc, and spray dried lactose to make the compression coat weight 200 mg.

**Table 3:** Physical properties of FLB-GG compression coated tablets.

Formulation	Weight variation* (mg)	Hardness^†^ (Kg/cm^2^)	Friability (%)	Drug content^‡^ (%)
F1	374.55 ± 3.36	6.43 ± 0.35	0.18	99.12 ± 1.23
F2	377.55 ± 3.43	6.37 ± 0.25	0.13	98.83 ± 1.46
F3	373.95 ± 3.30	6.21 ± 0.12	0.09	99.30 ± 2.00
F4	371.04 ± 4.63	6.29 ± 0.31	0.18	100.87 ± 1.15
F5	374.12 ± 3.68	6.41 ± 0.56	0.09	99.06 ± 0.55

*All values represent mean ± standard deviation, *n* = 20.

^†^All values represent mean ± standard deviation, *n* = 6.

^‡^All values represent mean ± standard deviation, *n* = 3.

**Table 4:** Release kinetics of FLB-GG compression coated tablets.

Formulation	Zero order		First order		Higuchi	
	*K*_0_ (mg/hr)	*R* ^2^	*K*_1_ (hr^−1^)	*R* ^2^	*K* (mg/hr^−1/2^)	*R* ^2^
F1	24.59	0.960	1.14689	0.920	44.28	0.823
F2	21.33	0.978	0.91199	0.898	44.06	0.854
F3	3.333	0.971	0.17503	0.808	16.70	0.872
F4	3.391	0.963	0.17733	0.797	17.05	0.872
F5	1.798	0.948	0.1566	0.755	9.246	0.897

*K*_0_: zero-order rate constant, *K*_1_: first-order rate constant, *K*: higuchi model rate constant, and *R* ^2^: correlation coefficient.

**Table 5:** Release kinetics of FLB-GG compression coated tablets.

Formulation	*K*	*n*	*R* ^2^	MDT	*T*10% (h)	*T*80% (h)
F1	1.4777	3.3104	0.920	2.35	1.6	4.8
F2	1.6240	2.8135	0.919	8.42	2.4	12.0
F3	1.0881	1.4983	0.951	10.34	3.8	14.6
F4	1.0954	1.1592	0.950	11.59	5.6	17.9
F5	1.3280	1.3370	0.928	13.71	5.8	24.0

*K*: kinetic rate constant, *n*: diffusional exponent, *R* ^2^: correlation coefficient, MDT: mean dissolution time, *T*10%: time to release 10% drug release, and *T*80%: time to release 80% drug release.

**Table 6:** Stability studies of FLB-GG compression coated tablets F4.

Time (h)	Before storage	After 6 months	*t*-test at 0.05 LS	Similarity Factor (F2)
0	0.00 ± 0.00	0.00 ± 0.00	Not significant	86.82
2	1.26 ± 0.12	1.20 ± 0.18		
4	1.97 ± 0.08	1.94 ± 0.01		
5	6.84 ± 0.09	6.25 ± 0.12		
8	42.64 ± 0.12	41.28 ± 0.14		
12	59.17 ± 0.36	58.93 ± 0.61		
18	82.62 ± 0.52	79.97 ± 0.26		
24	99.86 ± 0.75	99.43 ± 0.42		

% assay	100.87 ± 1.15	100.12 ± 0.86	Not significant	—

**Table 7:** Pharmacokinetics of FLB-GG colon specific and immediate release tablets.

Parameters (*n* = 6)	FLB colon specific tablets	FLB immediate release tablets	*P*
*C*_max⁡_ (ng/mL)	11956.15 ± 17.58	15677.52 ± 4.41	<0.05
*T*_max⁡_ (h)	10.00 ± 0.01	3.00 ± 0.01	*<*0.05
AUC_0–*∞*_(ng·h/mL)	78214.50 ± 132.15	40385.78 ± 96.11	*<*0.05
AUMC_0–*∞*_ (ng·h/mL)	782517.48 ± 1926.17	140857.33 ± 1361.29	*<*0.05
MRT (h)	10.78 ± 0.01	3.49 ± 0.03	*<*0.05
